# Irradiance of Horizontal Quartz-Halogen Standard Lamps

**DOI:** 10.6028/jres.101.016

**Published:** 1996

**Authors:** Edward A. Early, Ambler Thompson

**Affiliations:** National Institute of Standards and Technology, Gaithersburg, MD 20899-0001

**Keywords:** color temperature, lamp orientation, quartz-halogen lamps, spectral irradiance, standard lamps, ultraviolet

## Abstract

Spectral irradiance calibrations often require that irradiance standard lamps be oriented differently than the normal calibration orientation used at the National Institute of Standards and Technology and at other standards laboratories. For example, in solar measurements the instruments are generally upward viewing, requiring horizontal working standards for minimization of irradiance calibration uncertainties. To develop a working standard for use in a solar ultraviolet intercomparison, NIST determined the irradiance of quartz-halogen lamps operating in the horizontal position, rather than in the customary vertical position. An experimental technique was developed which relied upon equivalent lamps with independent mounts for each orientation and a spectroradiometer with an integrating sphere whose entrance port could be rotated 90° to view either lamp position. The results presented here are limited to 1000 W quartz-halogen type lamps at ultraviolet wavelengths from 280 nm to 400 nm. Sources of uncertainty arose from the lamps, the spectroradiometer, and the lamp alignment, and increased the uncertainty in the irradiance of horizontal lamps by less than a factor of two from that of vertical NIST standard lamps. The irradiance of horizontal lamps was less than that of vertical lamps by approximately 6 % at long wavelengths (400 nm) to as much as 12 % at the shortest wavelengths (280 nm). Using the Wien radiation law, this corresponds to color temperature differences of 15.7 K and 21.3 K for lamps with clear and frosted envelopes, respectively.

## 1. Introduction

The increased concern over stratospheric ozone depletion has prompted several Federal agencies to establish ground-based solar ultraviolet irradiance monitoring networks [1]. The instruments used at the sites of these networks are usually spectroradiometers with horizontal diffusers as their receiving apertures, i.e., the normal of the diffuser is vertical, so the irradiance from nearly the entire sky is measured. These instruments must be routinely calibrated using spectral irradiance standards to detect long-term trends in solar radiation and ozone depletion. To meet this requirement, lamps of known spectral irradiance operating in the horizontal position are needed so that the instruments can be calibrated in the position in which they are regularly used.

The primary standard for the spectral radiance scale maintained at the Facility for Automated Spectroradiometric Calibrations (FASCAL) at the National Institute of Standards and Technology is a blackbody operating at the freezing temperature of gold [2]. This scale is transferred via a variable temperature blackbody to a special integrating sphere to realize the spectral irradiance scale, which is maintained by a group of four, 1000 W, quartz-halogen modified FEL-type lamp secondary standards [3]. This group is used for routine calibrations of transfer standard FEL-type lamps available from NIST. Both the secondary and transfer standard lamps are calibrated by FASCAL in the vertical position, i.e., the long axis of the lamp filament is vertical.

The need for spectral irradiance standard lamps calibrated in the horizontal position was particularly acute at the first North American Interagency Inter-comparison of Ultraviolet Monitoring Spectroradiometers, held September 19 to 29, 1994 outside Boulder, Colorado, and organized by NIST and the National Oceanic and Atmospheric Administration (NOAA). Such lamps were necessary so that all participating instruments could be calibrated for spectral irradiance using a common standard. Therefore, two 1000 W quartz-halogen working standard lamps were calibrated in the horizontal position. This paper describes the issues and techniques involved in calibrating and using such lamps, and presents results on the changes in spectral irradiance from using the lamps in a horizontal rather than a vertical position.

## 2. Apparatus

### 2.1 Overview

**Fig. 1:**
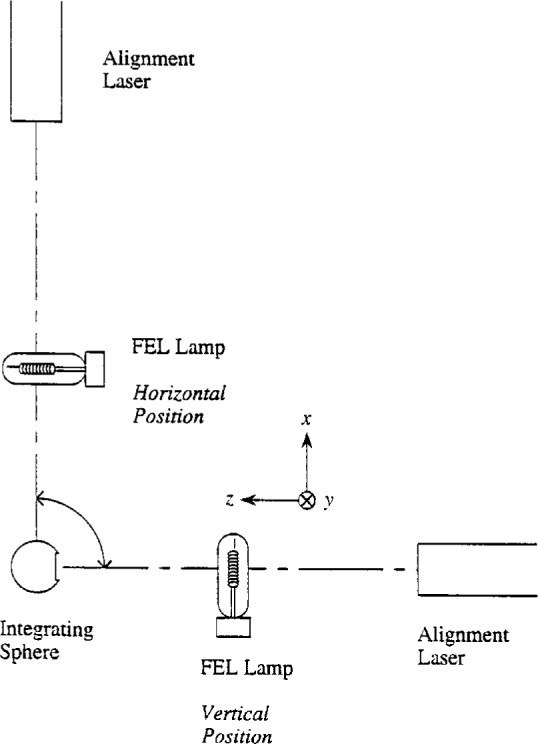
Schematic diagram of the positions of the integrating sphere, lamps, and alignment lasers. The integrating sphere could rotate by 90° to view either lamp position. The position nomenclature for each lamp is indicated, along with the coordinate directions for the vertical lamp.

The apparatus used for determining the spectral irradiance of lamps in a horizontal position consisted of those items necessary for generating, collecting, and measuring optical radiation, which is divided into light sources and the spectroradiometer. The sources consisted of FEL-type lamps with mounts, alignment lasers, and a power supply, while the spectroradiometer consisted of an integrating sphere, imaging optics, a double monochromator, and detectors. The features that were vital for performing the experiment were an integrating sphere whose entrance port could be rotated by 90°, and independent mounts for the vertical and horizontal lamps. The mounts for the integrating sphere, lamps, and alignment lasers were all on one optical table, while the imaging optics, monochromator, and detectors were on a separate platform, and the electronics were in separate racks. A schematic diagram illustrating the positions of the integrating sphere, lamps, and lasers is shown in Fig. 1, along with the coordinate directions for the vertical lamp.

### 2.2 Sources

The lamps were 1000 W quartz-halogen FEL-type with tungsten coiled-coil filaments. The lamp base had been converted to a medium bipost base, the structure of which was encapsulated in an epoxy-ceramic compound [3]. Three lamps, two with clear envelopes and one with a frosted enveloped and designated F-305, F-410, and WS-25, respectively, were calibrated as spectral irradiance secondary standards at FASCAL. Two other lamps, one clear and one frosted and designated OS-27 and F-45, respectively, were initially uncalibrated, and after calibration in the horizontal position were used as working standards in the Interagency Intercomparison. All the lamps had been checked for temporal stability and measured for goniometric distribution of irradiance prior to the measurements described here.

The current from the power supply to the lamps was controlled by an external voltage supplied by a computer-controlled 16 bit digital-to-analog converter. The current through the lamps was determined by measuring the voltage across a calibrated shunt resistor with a 6 1/2 digit voltmeter, and the current from the power supply was adjusted at intervals of 3 min to maintain the desired value. The voltage across the lamp was also monitored by the voltmeter. The details of the current control to the lamp are given in Ref. [4].

An alignment jig [3] was used to correctly position the lamp relative to the entrance port of the integrating sphere. The jig consisted of a medium bipost base identical to those of the FEL-type lamps, but with the posts extending above the base by 8.4 cm and filled in with an epoxy-ceramic compound. A 1.0 cm by 1.5 cm hole in the epoxy was centered between the posts and 9.5 cm above the ends of the posts. A glass plate was mounted over the hole so that one of its surfaces fell in the plane tangent to the surfaces of the posts. The center of the glass plate was located by scribe marks on the epoxy and a post, and the width of the entire jig at the location of the glass plate was 2.8 cm. The lamps and jig were mounted in specially designed lamp sockets that fixed the lamp height and to which the current and voltage leads were attached [2].

For the vertical lamps, a socket mounted on tilt and translation stages was attached to a platform approximately 50 cm from the integrating sphere and mounted on the optical table. Taking the center of the lamp as the origin, a Cartesian coordinate system is defined with the *x* axis along the long axis of the filament and the *z* axis along the line joining the lamp and the integrating sphere, as indicated in Fig. 1. The tilt stages permitted rotations of the lamp about the *x* and *y* axes, while the translation stages permitted translations along all three axes. A HeNe alignment laser attached to tilt and translation stages was mounted on the optical table 56 cm behind the lamp.

A heavy-duty tripod with a vertical post having a length of 80 cm was used to mount and align the horizontal lamps. A lamp socket was attached to brackets and tilt and translation stages that were clamped to the bottom of the post. As with the vertical lamp, these stages allowed rotations about two axes and translations along all three. A HeNe alignment laser 72 cm above the lamp was fixed in a tilt stage clamped to the top of the vertical post.

Rods were positioned to hold a shutter halfway between the integrating sphere and the lamps to block the direct path of light from the lamps, thereby allowing a measurement of the diffuse, scattered, light reaching the entrance port. The shutter itself was a black velveteen cloth wrapped around a cylinder with a 3 cm diameter, similar to that used by FASCAL. Black aluminum plates were positioned to block any reflections from the lamp sockets and the epoxy bases.

### 2.3 Spectroradiometer

The integrating sphere had an inside diameter of 2.54 cm and was coated with polytetrafluoroethylene (PTFE). The entrance and exit ports were 908 apart, and both had rectangular dimensions of 0.32 cm by 0.87 cm. A ring of diameter 1.43 cm was centered on the entrance port. The long axis of the entrance port was oriented so that this axis and the long axes of both lamp filaments defined a plane, as illustrated in Fig. 1. Therefore, the integrating sphere viewed both lamps in the same position relative to the entrance port. The integrating sphere was mounted on a platform on the optical table with tilt and translation stages. During measurements of lamps, a black cylinder of length 2.5 cm was attached to the ring to reduce the field of view of the entrance port to approximately twice the dimensions of the lamp.

One plane and one spherical mirror imaged the exit port of the integrating sphere onto a 1.0 mm wide by 0.8 mm high metal mask in front of the entrance slit of the monochromator. The prism-grating double monochromator had adjustable slit widths and a computer-controlled wavelength setting. A photomultiplier, a photodiode, and a HeNe laser were mounted behind the exit slit of the monochromator on a sliding platform so that each could be placed at the output of the monochromator. The laser defined the optic axis of the spectroradiometer. The photomultiplier tube (PMT) was a thermoelectrically cooled, magnetically shielded, end-on, trialkali tube operating in current mode. The photodiode was a specially selected Si photodetector. The current output of the PMT or photodiode was amplified and converted to voltage that was read by a 6 1/2 digit voltmeter. Additional details on the apparatus can be found in Ref. [5].

To reduce scattered light reaching the spectrora-diometer during measurements, black velveteen cloth was hung behind and to the sides of the lamps, on the optical table, and over the lasers and tripod.

## 3. Procedure

### 3.1 Spectroradiometer

#### 3.1.1 Wavelength Calibration of Monochromator

Because the wavelength setting of the monochromator recorded by the computer as encoder units did not correspond to the actual wavelength, a conversion factor was determined to convert from measured to actual wavelength. A Hg vapor lamp was placed next to the entrance port of the integrating sphere and the emission lines between 280 nm and 410 nm were scanned at 0.05 nm increments. The bandwidth (full width at half maximum) of the monochromator was 1.0 nm. The centroids of the lines were calculated in terms of measured wavelength and compared to the centroids based upon the actual wavelengths and intensities of the lines [6]. The differences between measured and actual centroids were least-squares fit with a second-order polynomial, which was used in subsequent experiments to convert measured to actual wavelength. The standard deviation of the fit yields a standard uncertainty of 0.02 nm in the wavelength of the spectroradiometer.

#### 3.1.2 Alignment of Integrating Sphere

The exit port of the integrating sphere should be centered on the optic axis defined by the monochromator and imaging optics so that rotations of the sphere do not change the area of the exit port imaged onto the metal mask. The beam from the HeNe laser located behind the exit slit of the monochromator was sent through the spectroradiometer to define the optic axis. The mount for the integrating sphere was adjusted with the tilt and translation stages so that the optic axis passed through the center of the mount. The integrating sphere was then placed in the mount so that the optic axis was centered on the exit port. Final adjustments were made with the stages so that approximately the same area was imaged onto the metal mask in front of the entrance slit of the monochromator upon rotation of the integrating sphere.

Because slightly different areas of the exit port were imaged upon rotating the integrating sphere, the effect of this on the measurements was determined. The output of a HeNe alignment laser was measured by placing the entrance port of a separate integrating sphere in the laser beam. This integrating sphere had a 2.5 cm inner diameter and was coated with PTFE powder. A Si photodiode was placed at the exit port of the integrating sphere to measure the output of the laser, and the current was amplified, converted to voltage, and read by the voltmeter.

The HeNe alignment lasers for both the vertical and horizontal lamp positions were aligned as described in Sec. 3.2 at the center and along the normal of the entrance port of the integrating sphere of the spectroradiometer. Using rods and clamps, the integrating sphere with the Si photodiode was then attached to one of the platforms used for the lamps. A glass microscope slide was placed over the entrance port of the sphere and the sphere was tilted and translated until the laser beam was centered on the port and the retroreflection from the slide returned along the laser beam. The glass slide was removed and the signal from the photodiode was measured for 1 min. The integrating sphere was then removed, allowing the laser beam to fall on the entrance port of the integrating sphere of the spectroradiometer. With the wavelength of the monochromator set at 632.8 nm and a bandwidth of 6 nm, the signal from the Si photodiode at the exit slit of the monochromator was measured for 1 min. The integrating sphere was then replaced and realigned, and the signal was measured for 1 min. This served to bracket the signal from the spectroradiometer by measurements of the output of the HeNe laser, which normalized the signal measured by the spectroradiometer. This sequence was repeated for the HeNe laser used for the other lamp position.

### 3.2 Alignment of Lamps

The goal in aligning the lamps was to position the center of the lamp 50.0 cm from, and perpendicular to the normal of, the entrance port of the integrating sphere. This was accomplished in two steps using the HeNe alignment laser. The first step was to locate the normal of the entrance port. A glass microscope slide was placed on the ring around the entrance port, and the laser beam was reflected off the slide. The tilt and translation stages of the laser were adjusted until the beam was located at the center of the entrance port and was retroreflected back to the laser. This defined the normal based upon the laser beam, and the laser was not adjusted after this step.

The second step was to position the lamp jig. The jig was inserted into the lamp socket and rotated about the *x* and *y* axes so that the laser beam was retroreflected. The jig was then translated along the *x* and *y* axes so that the laser beam passed through the center of the window, as determined from the scribe marks on the jig. Finally, the jig was translated along the *z* axis so that the edge nearest the integrating sphere was 50.0 cm from the entrance port, as determined by a stick marked with the appropriate distance. The adjustment of translations and tilts were repeated until all three conditions were met: (1) jig perpendicular to normal, (2) jig centered on normal, and (3) edge of jig 50.0 cm from entrance port.

### 3.3 Measurement Sequence

For a sequence of lamp measurements on a given day, both the horizontal and vertical lamp positions were aligned using the procedure given above. A measurement of a single lamp consisted of placing the lamp in the appropriate position, rotating the integrating sphere to view that lamp, and placing the shutter between the lamp and the sphere. The current was then ramped up to the operating value for each lamp: 7.8 A for F-305, 8.0 A for WS-25 and F-45, and 8.2 A for F-410 and OS-27. A system shutter scan from 405 nm to 275 nm at 5 nm intervals was performed, taking three readings from the PMT detector at each wavelength to measure the diffuse signal. Upon completion of this scan, the shutter was removed and the scan was repeated as before, this time measuring the total signal. The time required for these two scans was approximately 30 min. The bandwidth of the monochromator for all lamp measurements was 8 nm, and the room lights were turned off during all scans.

Measurements of lamp irradiances, either in the vertical or the horizontal position, were bracketed by measurements of lamps with known irradiances in the vertical position. These latter measurements served to determine the responsivity of the spectroradiometer. The time interval between measurements was kept as short as possible within the constraints of switching current leads, inserting lamps, and waiting for lamps to cool. The sequence of lamp measurements for each day, indicating the lamp and its position, is given in Table 1. After the lamps were measured on day 1, the lamp power supply and the entire apparatus used to mount and align the horizontal lamps were taken down and used at the Interagency Intercomparison near Boulder, Colorado to calibrate the ultraviolet network spectroradiometers. At the Intercomparison, lamps OS-27 and F-45 were operated for 12 h and 2.5 h, respectively. The apparatus was then reassembled at NIST, Gaithersburg for the measurements on days 2, 3, and 4.

## 4. Results

### 4.1 Calculations

The principal differences between measurements were the lamps and their orientations, as well as slight differences resulting from alignments. Since the spectroradiometer position, bandwidth, and detector were not changed, and the lamps had approximately the same spectral shape and irradiances, it is permissible to use the simplified measurement equation from Ref. [7],

S(λ)=E(λ)R(λ) (1)

Here, *S* (*λ*) is the signal measured by the detector of the spectroradiometer, *E* (*λ*) is the spectral irradiance of the lamp, and *R* (*λ*) is the spectral irradiance responsivity of the spectroradiometer. For simplicity, the term “responsivity” is used throughout this paper in place of the more descriptive term “spectral irradiance responsivity.”

The calculation of the lamp irradiance began by converting measured wavelengths to actual wavelengths using the calibration detailed in Sec. 3.1.1. The three diffuse and total signals at each wavelength were averaged, and the former was subtracted from the latter to obtain the direct signal. The direct signals were interpolated using a natural cubic spline fit to wavelengths from 280 nm to 400 nm at 5 nm intervals.

The lamps calibrated by FASCAL in the vertical position were used to determine the responsivity of the spectroradiometer. From Eq. (1), the responsivity is simply the direct signal divided by the lamp irradiance. Because the lamp irradiances from FASCAL are given at 10 nm intervals, they were interpolated using a natural cubic spline fit to the same wavelength range and interval as the signals. The irradiance of a lamp is, from Eq. (1), the direct signal divided by the responsivity. For these calculations, the responsivity was taken to be the average responsivity determined from the two bracketing measurements.

The Wien radiation law for radiance as a function of wavelength and temperature was used to calculate lamp color temperatures based upon their measured spectral irradiance. This expression is given by

$$E\left(\lambda ,T\right)=\frac{\Omega \varepsilon c_{1}\exp[-c_{2}/n\lambda T]}{\pi n^{2}\lambda^{5}}$$ (2)

where *Ω* is the solid angle from the lamp to the entrance port of the integrating sphere, *ε* is the effective emissivity, *c*_1_ = 1.191 062 × 10^20^ W m^2^ is the first radiation constant, *c*_2_ = 1.438 786 × 10^7^ K nm is the second radiation constant, *n* is the index of refraction, *λ* is the wavelength, and *T* is the temperature.

### 4.2 Uncertainties

A detailed description and analysis of the uncertainties, particularly with regard to the alignment of the lamps, is given here in anticipation of its applicability to irradiance calibration results from the Interagency Ultraviolet Spectroradiometric Intercomparison. The sources of uncertainty in the calculated responsivities and irradiances were the lamps, spectrometer, and alignment of the lamps with the integrating sphere. Much of the analysis of these uncertainties relies upon the geometrical throughput of the system consisting of the lamp and the entrance port of the integrating sphere [8]. An extended source (lamp) and detector (entrance port) have areas *A*_s_ and *A*_d_, respectively. The distance between the centers of the areas is *D*, and the normal of the detector makes an angle *θ* with respect to the line joining the centers of the areas. For *D* much greater than the largest linear dimension of the detector and source, and assuming that the source is spherically symmetric, the throughput *Γ* of the system is given by

$$\Gamma =\frac{A_{s}A_{d}}{D_{2}}\cos\theta .$$ (3)

The power at the detector, *ϕ*_d_, is given by *L*_s_*Γ*, where the radiance of the source *L*_s_ is assumed to be uniform over the source area. Thus, the irradiance at the detector, *E*_d_, is given by

$$E_{d}=\frac{\phi_{d}}{A_{d}}=\frac{L_{s}}{A_{d}}\Gamma .$$

Given that the irradiance of the source is proportional to its radiance, this yields a relation for the irradiance at the detector in terms of the irradiance of the source and the throughput of the system, namely

$$E_{d}\propto \frac{E_{s}}{A_{d}}\Gamma .$$ (4)

Since the areas of the source and detector do not change, the sources of uncertainty in *E*_d_ are, from Eqs. (3) and (4), the irradiance of the source *E*_s_, the distance *D*, and the angle *θ*.

In evaluating, propagating, and reporting the uncertainties involved in the various calculations, the guidelines and nomenclature given in Ref. [9] are used. There are both Type A uncertainties (those evaluated using statistical methods) and Type B uncertainties (those evaluated by other methods) present in the measurements. For Type B uncertainties, the probability distribution determines the standard uncertainty based upon the upper and lower limits of the distribution, as detailed in Ref. [9]. For a rectangular distribution, the half-width of the distribution is divided by $\sqrt{3}$ to obtain the standard uncertainty component, while for a triangular distribution the divisor is $\sqrt{6}$. All expanded uncertainties use a coverage factor of *k* = 2. The components of uncertainty are divided between those arising from the lamps, the spectroradiometer, and alignment of the lamps.

#### 4.2.1 Lamps

Using the lamp current control method detailed in Ref. [5], the standard uncertainty in current is *u* (*I*) = 0.2 mA. The relative uncertainty in the irradiance of the lamp is given by [10]

$$\frac{u\left(E\right)}{E}=\left(\frac{654.6}{\lambda /\text{nm}}\right)(0.000\;6)(u(I)/\text{mA}).$$ (5)

This is a Type B relative standard uncertainty based on an assumed normal probability distribution since the primary source of uncertainty is the resistance of the shunt used to measure the current.

The relative standard uncertainty in lamp irradiance is given by the FASCAL calibration at selected wavelengths. These values were interpolated using a polynomial fit to wavelengths between 280 nm and 400 nm, inclusive, at 5 nm intervals. For the purposes of the calculations in this paper, these uncertainties are Type B based on an assumed normal probability distribution even though they were obtained by statistical methods by FASCAL [9].

#### 4.2.2 Spectroradiometer

The standard uncertainty in the average signal is simply the standard deviation of the mean of the three signals used for the average. These uncertainties are propagated through the calculation of the direct signal. The standard uncertainty at an interpolated wavelength is the uncertainty at the actual wavelength closest to the interpolated wavelength. The relative standard uncertainty in the direct signal is the standard uncertainty divided by the signal, and is obviously Type A.

As stated in Sec. 3.1.1, the standard uncertainty in wavelength of the monochromator is *u* (λ) = 0.02 nm. This corresponds to a relative standard uncertainty in irradiance given by

$$\frac{u(E)}{E}=\left(\frac{\lambda }{E}\right)\left(\frac{\partial E}{\partial \lambda }\right)\left(\frac{u(\lambda )}{\lambda }\right).$$ (6)

The spectral irradiances *E* (*λ*) of the lamps calibrated by FASCAL were fit with third-degree polynomials to obtain *δE* / *δλ*. Equation (6) was then used to calculate the relative uncertainty, which is Type B uncertainty based on an assumed normal probability distribution.

The uncertainty resulting from the orientation of the integrating sphere was measured using the procedure given in Sec. 3.1.3. For each orientation of the integrating sphere, the average signal from the spectroradiometer Si photodiode was divided by the average signal from the monitoring Si photodiode. The standard uncertainty in the resulting value was obtained from the standard deviation of the mean of the signals. Taking the ratio of the normalized signal with the integrating sphere viewing the vertical lamp position to that for the horizontal lamp position determined on three separate occasions, and propagating uncertainties, yields values and standard uncertainties of 1.001 03 ± 0.000 67, 0.998 251 ± 0.003 715, and 0.997 951 ± 0.002 691. Thus, the mean ratio differs from unity by at most 0.002, which for a rectangular probability distribution yields a Type B relative standard uncertainty of 0.0012 in the irradiance for lamps measured in the horizontal position.

#### 4.2.3 Alignment

Six components of uncertainty are present from aligning the center of the lamp 50 cm from and perpendicular to the normal of the entrance port of the integrating sphere. A tilt of the entrance port results in an angular uncertainty in the normal direction, while a tilt in the lamp alignment jig causes an angular uncertainty between the lamp and the entrance port. Likewise, uncertainties in locating the centers of the entrance port and the alignment jig result in an angular uncertainty between the lamp and the entrance port. These three components of uncertainty combine with the goniometric distribution of irradiance of the lamp to give an uncertainty in the lamp irradiance. Uncertainties in the distance between the alignment jig and the entrance port arise from aligning the jig with the mark on the stick and from the placement of the stick with respect to the entrance port. The evaluation of each component of uncertainty is discussed in the following paragraphs.

The uncertainty in defining the normal of the entrance port of the integrating sphere is given by the displacement of the retroreflection of the laser beam from the entrance port. The maximum displacement was ±1.0 cm, which for a Type B uncertainty based on a rectangular probability distribution yields a standard uncertainty of 0.58 cm. The standard uncertainty in the angle of the normal *u* (*θ*) is this displacement divided by the distances between the entrance port and the lasers, which are 106 cm and 122 cm for the vertical and horizontal lamp positions, respectively. This yields *u* (*θ*) = 0.0054 rad and 0.0047 rad for the two lamp orientations. Using a Taylor series expansion of the throughput given by Eq. (3) results in relative standard uncertainties in the irradiance of 0.000 025 and 0.000 019 for the vertical and horizontal lamp positions, respectively.

The uncertainty in aligning the lamp jig perpendicular to the normal of the entrance port is given by the displacement of the retroreflection from the glass window. The maximum displacement was ±2.0 mm, which for a Type B uncertainty based on a rectangular probability distribution yields a standard uncertainty of 1.2 mm. The standard uncertainty in angle *u* (*θ*) is this displacement divided by the distances between the jig and the lasers, which are 56 cm and 72 cm for the vertical and horizontal lamp positions, respectively. This yields *u* (*θ*) = 0.0021 rad and 0.0016 rad for the two lamp orientations. The maximum displacement of the laser beam from the center of the entrance port was ±1.0 mm in either orthogonal direction, yielding a standard uncertainty of 0.82 mm. For the center of the glass window on the lamp jig, the maximum displacement was ±0.5 mm, resulting in a standard uncertainty of 0.4 mm. The corresponding angular standard uncertainties using a distance of 50 cm between the jig and the entrance port are *u* (*θ*) = 0.0016 rad and 0.0008 rad, respectively. Combining the angular standard uncertainties in quadrature yields *u* (*θ*) = 0.0028 rad and 0.0025 rad for the vertical and horizontal lamp positions, respectively.

The goniometric distribution of irradiance refers to the dependence of lamp irradiance on the angle at which the lamp is viewed. Possible causes for this distribution are shadowing of one coil by another and non-uniformity in the transmission of the lamp envelope. The goniometric distribution is measured by FASCAL at one wavelength as a ratio of the lamp irradiance at pitch and yaw angles of ±1.5° at intervals of 0.5° to the irradiance when the lamp is properly oriented [3]. The goniometric distributions of lamps F-305, WS-25, and F-45 were measured at 300 nm, while those of lamps F-410 and OS-27 were measured at 654.6 nm. It is assumed that the goniometric distributions are independent of wavelength. The root-mean-square values of the goniometric distributions within 0.5° of 0° are *g* = 1.0027 for lamp F-305, 1.001 for lamp F-410, 1.0029 for lamp OS-27, 1.0019 for lamp WS-25, and 1.0024 for lamp F-45. The relative standard uncertainty in the irradiance resulting from the above angular standard uncertainties in the preceding paragraph are thus given by

$$\frac{u(E)}{E}=\left(\frac{u(\theta )}{0.5^{\circ }}\right)(1-g).$$ (7)

For example, this results in a relative standard uncertainty for lamp F-410 in the vertical position of 0.00032.

The relative standard uncertainty in irradiance due to a standard uncertainty *u* (*D*) in the distance from the center of the entrance port to the center of the lamp jig is obtained by differentiating Eq. (3) with respect to *D* and is given by

$$\frac{u(E)}{E}=\frac{2u(D)}{D}.$$ (8)

The maximum displacement in aligning the 50 cm mark on the stick with the bottom of the lamp jig was ±1.0 mm. This results in a Type B uncertainty based on a triangular probability distribution, so *u* (*D*) = 0.41 mm. Using Eq. (8), the relative standard uncertainty in irradiance *u* (*E*)/*E* = 0.0016. The maximum displacement of the stick with respect to the center of the entrance port is the edge of the surrounding ring, a distance of 0.28 cm. Thus, assuming a triangular probability distribution, the standard uncertainty in measuring from the center of the entrance port is 0.12 cm. This yields a standard uncertainty $u(D)=50-\sqrt{{\left(50\right)}^{2}-{\left(0.12\right)}^{2}}=0.000$ 14 cm, giving a relative standard uncertainty in the irradiance *u* (*E*)/*E* = 0.000 005. The combined relative standard uncertainty due to the distance is *u* (*E*)/*E* = 0.0016.

#### 4.2.4 Summary

The components of uncertainty are divided between those arising from the lamps, the spectroradiometer, and the alignment of the lamps with respect to the spectroradiometer. There is uncertainty in the current through the lamp and the irradiance of the lamp; in the signal from the detector, the wavelength of the monochromator, and the orientation of the integrating sphere; and in the normal of the entrance port, aligning the lamps perpendicular to this normal, aligning the centers of the entrance port and the lamps, and the distance between the entrance port and the lamps.

**Fig. 2:**
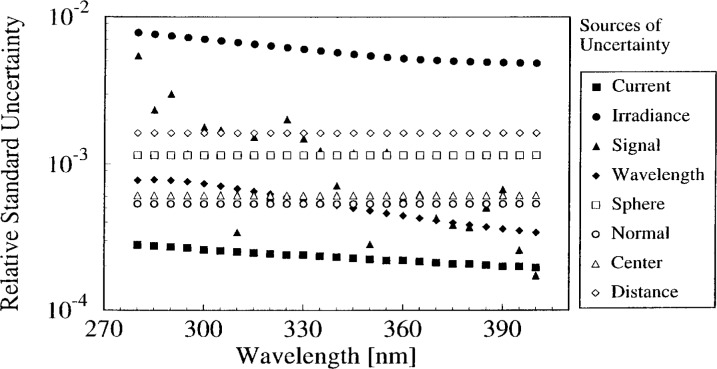
Relative standard uncertainty components of irradiance as a function of wavelength from the indicated sources. These sources are the current through the lamp, irradiance of the lamp, signal from the detector, wavelength accuracy of the monochromator, integrating sphere orientation, and alignment of the lamp normal to and centered on the normal of the entrance port at the specified distance.

The relative standard uncertainties in irradiance *u* (*E*)/*E* that result from these uncertainties are shown in Fig. 2, where *u* (*E*)/*E* is plotted as a function of wavelength for representative data. The largest uncertainty is the irradiance of the vertical lamps, while the second largest at all wavelengths is the distance between the lamp and the integrating sphere. The relative standard uncertainties arising from the alignment of the lamps span the range from 1.6 × 10−^3^ to 5.4 × 10−^5^. The uncertainty from the signal decreases from the second largest at short wavelengths to the second smallest at long wavelengths. In terms of potential improvements, the uncertainties from the spectroradiometer (signal, wavelength, and sphere orientation) could be decreased by an improved design. The uncertainty from the alignment would be improved by decreasing that arising from the distance between lamp and integrating sphere. However, to obtain a dramatic improvement in the uncertainty, a decrease in the uncertainty of the calibrated transfer standard vertical lamps is absolutely essential.

### 4.3 Spectroradiometer Stability

**Fig. 3:**
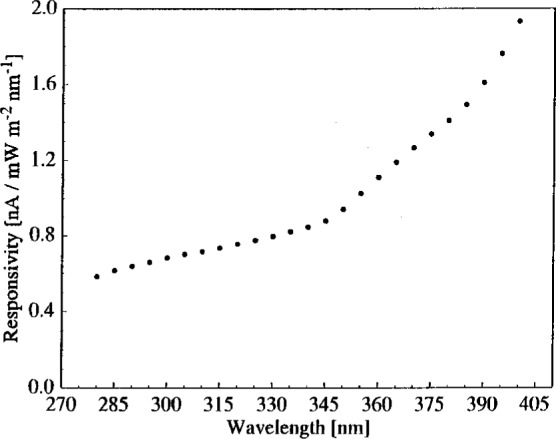
Responsivity of the spectroradiometer as a function of wavelength as determined by lamp F-410 on day 2. The expanded combined standard uncertainties (coverage factor *k* = 2) are comparable to the sizes of the symbols.

The responsivity *R* is calculated for each calibrated lamp measured in the vertical position. A representative result is shown in Fig. 3, where the responsivity is plotted as a function of wavelength for lamp F-410 measured on day 2. The responsivity increases relatively smoothly with increasing wavelength, as expected for the type of PMT used as the detector. The expanded uncertainties of the responsivity are the sizes of the symbols in Fig. 3, and arise from all of the components given in the previous section except that from the sphere orientation.

**Fig. 4:**
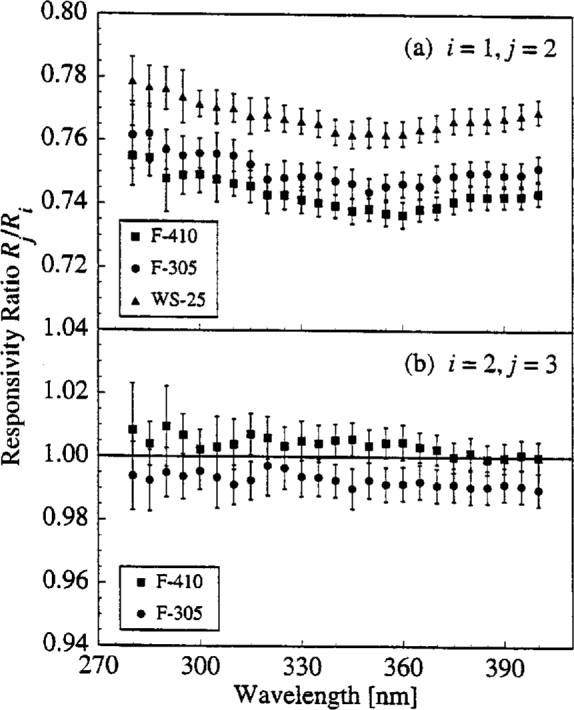
Responsivity ratio *R_j_*/*R_i_* between days as a function of wavelength for the indicated lamps. The days used in the ratios are given by the indices *i* and *j*. The vertical bars indicate the expanded combined standard uncertainty (coverage factor *k* = 2) of the ratios.

The stability of the spectroradiometer is given by the ratios of responsivities *R_i_* determined on different days *i* for the same lamps. These are shown in Fig. 4, where the ratio of the responsivities for each lamp is plotted as a function of wavelength for (a) day 2 to day 1 and (b) day 3 to day 2. The expanded combined standard uncertainties are shown for each ratio as vertical bars, and result from uncertainty components due to the lamp currents, detector signals, and lamp alignments. Uncertainties from the lamp irradiances, monochromator wavelength, and sphere orientation are not included since none of these components changed from one measurement to the next.

The changes in the responsivity ratios between days and lamps are divided into those that are wavelength-independent and those that are wavelength-dependent. From Fig. 4(a), there is a wavelength-independent decrease in responsivity for all the lamps of approximately 23 % from day 1 to day 2, while from Fig. 4(b) there is essentially no wavelength-independent change for either lamp from day 2 to day 3. Since the responsivity ratios are not constant with respect to wavelength, there is also a wavelength-dependent change in responsivity between different days. This change is consistent between lamps in Fig. 4(a) and, while different in Fig. 4(b), is still consistent between lamps.

Since the responsivity ratios of lamps F-410 and F-305 agree with each other within their respective standard uncertainties, the significant change in spectroradiometer responsivity between day 1 and day 2 is not due to the lamps. In fact, the relative change in irradiance of lamp F-305 was only about 1 % over 2 years, even after suffering abuses in that time (such as sudden changes in current) that were not present for the measurements presented here. Therefore, the wavelength-independent change in responsivity shown in Fig. 4(a) is due to changes in the spectroradiometer, most likely in the detector, which is especially plausible given the long time interval between the days on which the measurements occurred. When the time interval is short, as in Fig. 4(b), the wavelength-independent relative change in responsivity was less than 1 %. The most plausible explanation for the disagreement between the responsivity ratio of lamp WS-25 and those of the other 2 lamps is that this lamp is more variable, as evidenced by a change in voltage from 110.5 V to 111.2 V between the two days. Since the wavelength-dependent changes in responsivity are consistent between lamps in both Figs. 4(a) and 4(b), these changes are also due to the spectroradiometer. If instead the lamps were varying, the changes would not be expected to be the same for both lamps.

**Fig. 5:**
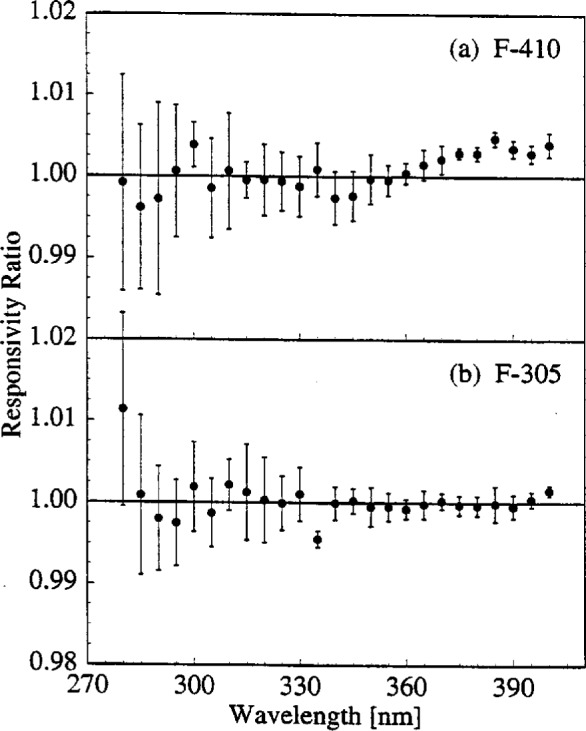
Responsivity ratio as a function of wavelength for two determinations with (a) lamp F-410 on day 3 and (b) lamp F-305 on day 4. The vertical bars indicate the expanded combined standard uncertainty (coverage factor *k* = 2) of the ratios.

This is further demonstrated in Fig. 5, where the responsivity ratios for lamps (a) F-410 and (b) F-305 are shown for measurements done on the same day, day 3 and day 4, respectively. The expanded combined standard uncertainties, shown for each ratio as vertical bars, arise from components due to the detector signals. A component from lamp alignment is not included since the alignment was not changed between measurements of the same lamp. The responsivity is unchanged between the two determinations with lamp F-305, and changes slightly with lamp F-410 for wavelengths longer than 370 nm.

The responsivity ratios indicate that the irradiances of the calibrated vertical lamps are constant between measurements, although their absolute values are uncertain. This conclusion will be applied in the succeeding results, as it was above in the uncertainty analysis. On the other hand, the responsivity of the spectroradiometer changes both between days and between measurements on the same day. Thus, it was necessary to determine the responsivity on each day and to bracket measurements of unknown lamp irradiances with determinations of responsivity, as was done in the calculations detailed in Sec. 4.1.

### 4.4 Alignment Reproducibility

**Fig. 6:**
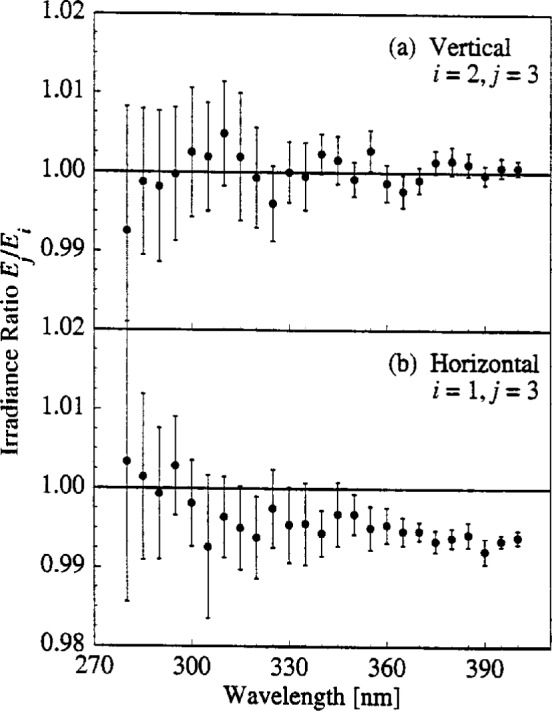
Irradiance ratio *E_j_*/*E_i_* between days as a function of wavelength for lamp OS-27 in the (a) vertical and (b) horizontal position. The days used in the ratios are given by the indices *i* and *j*. The vertical bars indicate the expanded combined standard uncertainty (coverage factor *k* = 2) of the ratios without including the uncertainty component arising from alignment of the lamps.

The ability to align the lamps reproducibly is demonstrated by the ratios of irradiance *E_i_* for lamp OS-27 determined in both the horizontal and vertical positions on different days *i*. The ratio of the irradiance measured on one day to that on another day is plotted in Fig. 6 as a function of wavelength for lamp OS-27 in the (a) vertical and (b) horizontal position. In the vertical position, the ratio is between day 3 and day 2, while in the horizontal position it is between day 3 and day 1. The expanded standard uncertainty includes components from only the detector signals. The other components are not included since they were unchanged between the measurements used in the ratios, except those due to lamp alignment. These are not included to demonstrate the alignment reproducibility that is possible. When they are included, the vertical bars in Fig. 6 are significantly longer and all of them overlap the horizontal line at a ratio of 1.00.

An irradiance ratio in Fig. 6(a) of 1.00, within the uncertainties, indicates that very good lamp alignment reproducibility was achieved for the vertical lamp position. This is not surprising, as this alignment was easier to achieve than for horizontal lamps since all of the platforms were attached directly to the optical table, and the measurements were performed only one day apart. The deviation of the irradiance ratio in Fig. 6(b) from 1.00, however, is due both to the greater difficulty in aligning this lamp position since a movable tripod was used, and to the longer time interval between the two measurements. Even so, the ratio is within 1 % of 1.00, which is comparable to the combined relative standard uncertainty for this lamp due to alignment, which is 0.87 %.

The results shown in Fig. 6 indicate that good lamp alignment reproducibility can be achieved since the deviations of the ratios from 1.00 are generally within the expanded combined standard uncertainties from other components. Including the component from alignment brings the ratios into agreement with 1.00. These results also show that there is no effect from operating a lamp in both positions, since the irradiance in one position remained unchanged upon operating the lamp in the other position.

### 4.5 Horizontal Irradiances

**Fig. 7:**
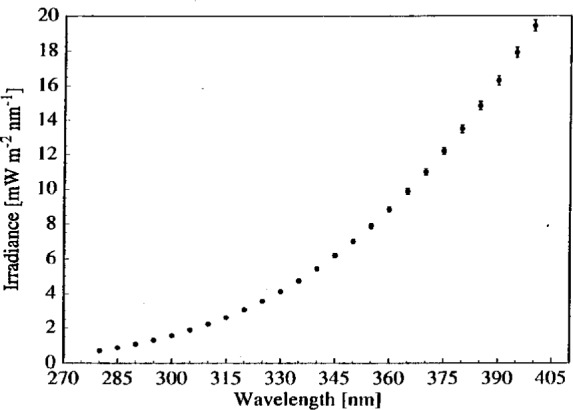
Irradiance as a function of wavelength for lamp OS-27 in the horizontal position. The vertical bars indicate the expanded combined standard uncertainty (coverage factor *k* = 2) of the irradiance.

Lamp OS-27 was used in the Interagency Intercomparison to calibrate all of the participating spectroradiometers. The irradiance of this lamp in the horizontal position as a function of wavelength is shown in Fig. 7, and is the average of the irradiances determined on days 1 and 3. The vertical bars indicate the expanded combined standard uncertainty of the irradiance. In comparison with the uncertainties given by FASCAL for vertical lamps, the measurements detailed in this paper increased the expanded relative standard uncertainty in irradiance from 1.57 % to 2.56 % at 280 nm and from 0.97 % to 1.65 % at 400 nm.

**Fig. 8:**
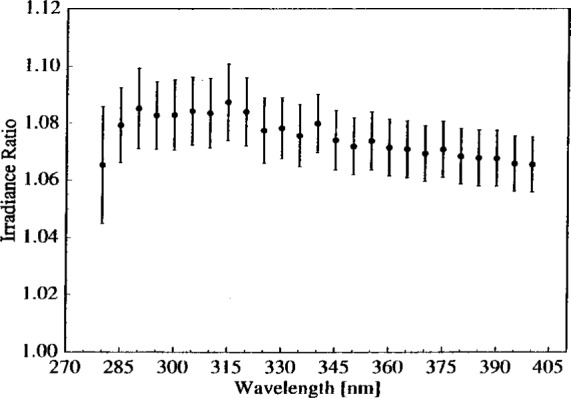
Ratio of vertical to horizontal irradiance as a function of wavelength for lamp OS-27 determined on day 3. The vertical bars indicate the expanded combined standard uncertainty (coverage factor *k* = 2) of the ratio.

The ratio of lamp irradiance in the vertical position to that in the horizontal position indicates the changes that result from the different lamp orientations. A typical example of such a ratio is shown in Fig. 8, where the irradiance ratio for lamp OS-27 between the vertical and horizontal positions on day 3 is plotted as a function of wavelength. The expanded combined standard uncertainties, shown as vertical lines, have components from the lamp currents, detector signals, integrating sphere orientation, and lamp alignments. The irradiance in the vertical position is about 6.5 % greater than it is in the horizontal position at the longest wavelengths. This difference increases to about 8.5 % as the wavelength is reduced, and then decreases at wavelengths less than 290 nm. These values and behavior are similar to other ratios using lamp OS-27 and to the one ratio for lamp F-305, the other lamp with a clear envelope. Because of the decrease at the shortest wavelengths, all fits detailed below were performed for wavelengths ≥300 nm. A study similar to this one performed on FEL-type lamps operated in the vertical position and at an angle of 62.5° from the horizontal shows similar behavior but with reduced values [11]. The ratios for lamp F-45, the one with a frosted envelope, increase from about 8 % to about 12 % with decreasing wavelength, with no decrease at the shorter wavelengths.

The color temperature of the vertical lamps was obtained from their spectral irradiances using the Wien radiation law, Eq. (2). The slope from a least-squares linear fit of ln{(*λ*/nm)[*E*/(W m^−2^ nm^−1^)]} vs 1/(*λ*/nm) yields the color temperature *T*. The uncertainties in the irradiance propagated through as uncertainties in the color temperature. The temperatures of the lamps in the vertical position, *T*_V_, are given in the second column of Table 2. The color temperatures of the lamps with a clear envelope are both approximately 3050 K, despite a difference in current of 0.4 A between them, while the temperature of the lamp with the frosted envelope is only about 2980 K.

The color temperature of a lamp in the horizontal position, *T*_H_, was determined from the irradiance ratio between the vertical and horizontal positions, *E*_V_/*E*_H_. From Eq. (2), this ratio is given by

$$\frac{E_{V}}{E_{H}}=\exp\left[\frac{-c_{2}}{\lambda }\left(\frac{1}{T_{V}}-\frac{1}{T_{H}}\right)\right].$$

Therefore, knowing the value of *T*_V_ from the analysis detailed in the preceding paragraph and performing a least-squares linear fit of ln(*E*_V_/*E*_H_) vs 1/*l* yields *T*_H_. The uncertainties in the irradiance ratio and in *T*_V_ are propagated through to give an uncertainty in *T*_H_. The resulting color temperatures for lamps in the horizontal position are given in the third column of Table 2. The last column in Table 2 gives the temperature difference for each lamp between the vertical and horizontal positions. The expanded combined standard uncertainty of the temperature difference is comparable to its value because the difference is between two large numbers.

The temperature differences listed in Table 2 show that the lamps with clear envelopes, OS-27 and F-305, even though from two different manufacturers, have a 15.7 K greater color temperature in the vertical position than in the horizontal position, while the lamp with the frosted envelope, F-45, has a 21.3 K greater temperature. It is unlikely that a physical change in the lamp filament is responsible for such a large temperature difference. The filament could sag under the influence of gravity differently when the lamp is in a horizontal position than in a vertical position. However, this is not a plausible explanation for the temperature difference because the lamp spectral irradiance in the vertical position does not change after having operated the lamp in the horizontal position, as demonstrated by lamps OS-27 and F-305. Furthermore, sagging of the filament in the horizontal position would bring it closer to the integrating sphere, with a corresponding increase in irradiance compared to the vertical position, not the decrease that is observed.

While the voltage across the lamp is slightly different between the two orientations, it is not consistent with the temperature difference. In the horizontal position, the voltages across lamps OS-27 and F-305 were 105.4 V and 110.4 V, respectively, while in the vertical position these voltages were 105.8 V and 110.5 V. There is an empirical relation between color temperature and voltage [12], (*T*_V_/*T*_H_) = (*V*_V_/*V*_H_)^0.42^, where the subscripts indicate the lamp position. Using the voltages given above and the vertical temperatures given in Table 2 yields temperature differences of only 5.0 K and 1.2 K for lamps OS-27 and F-305, respectively, which are significantly less than the actual values.

A plausible explanation for the large color temperature difference is the relative efficiency of heat transfer for the two orientations. However, other effects such as thermal conduction through the base of the lamp, may have an important role. Thus, additional research needs to be performed to determine the complete explanation. In terms of heat transfer, electrical power is supplied to the lamp filament and converted into heat, which is then transferred away from the filament by radiation, conduction, and convection. Most of the radiative power is transmitted through the quartz envelope with little absorption, except that at infrared wavelengths greater than about 3 μm. This, together with conduction and convection from the filament, heats the envelope, which in turn transfers the heat both back to the filament and out to the surroundings. Thus, the lamp filament and envelope reach steady-state temperatures determined by the different heat transfer processes. From the lamp voltages, the relative change in the power input to the filament between the two lamp orientations is less than 0.4 %. However, the process of convective heat transfer from the envelope to the surroundings is more efficient in the horizontal position than in the vertical position because of the larger horizontal surface area. Therefore, the envelope and filament are at lower temperatures for a horizontal lamp than for a vertical lamp, resulting in a lower ultraviolet irradiance. These concepts also explain why the color temperature of the frosted lamp is less than for the clear lamps. The frosting increases the emissivity of the envelope, thereby increasing the amount of power radiating from the envelope and consequently lowering its steady-state temperature and that of the filament.

## 5. Conclusions

There is an increasing need for irradiance standards operating in the horizontal position, as exemplified by the Interagency Ultraviolet Spectroradiometer Intercomparison. In response to this need, experimental techniques were developed to determine the irradiance of horizontal lamps. This was accomplished by extending the procedures involved in calibrating vertical lamps by FASCAL to include mounts for both vertical and horizontal lamps and an integrating sphere that could be rotated to view both lamp orientations. Sources of uncertainty in the measurements were the lamps, spectroradiometer, and alignments. The irradiance of lamps in the horizontal position was less than the irradiance for the same lamps in the vertical position by 6 % to 12 % of the latter, depending on the lamp and wavelength, which corresponds to color temperature differences of 15 K and 21 K for lamps with clear and frosted envelopes, respectively. The most likely explanation for the lower irradiance in the horizontal position is improved convective heat transfer from the envelope in this orientation.

The techniques presented here are generally applicable to irradiance calibrations of a variety of lamps in the horizontal position at all optical wavelengths. The discussion was limited to quartz halogen lamps at ultraviolet wavelengths only because there was an immediate need for such measurements. The key issues that were considered and addressed in these measurements were reproducing, as much as possible, the conditions under which vertical lamps are calibrated by FASCAL; using the same type of lamp for determining the responsivity of the lamp in the horizontal position; mounting and carefully aligning both vertical and horizontal lamps in the same manner; maintaining identical conditions for the spectroradiometer, except for the integrating sphere orientation, for measurements of both lamp orientations; and bracketing measurements of horizontal lamp irradiances with determinations of responsivity based upon vertical lamps. All of these considerations made it possible to determine horizontal lamp irradiances with expanded standard uncertainties that were only about 1.7 times larger than those of vertical lamps calibrated by FASCAL. Incremental reduction of the uncertainty of the irradiance of horizontal lamps will be possible through better spectroradiometer design and stability and by better alignment techniques. However, a substantial improvement is possible only by a significant reduction in the uncertainty associated with the calibration of vertical lamps. The relatively large decrease in irradiance of lamps in the horizontal position compared to the vertical position illustrates the critical need to calibrate these lamps in the orientation in which they will be used to determine the responsivity of spectroradiometers. The role of heat transfer in determining the irradiance of a lamp indicates that the environmental conditions under which the spectroradiometer is calibrated with the lamp may need to be controlled or taken into account.

## Figures and Tables

**Table 1:** Lamp measurement sequence. The lamps were measured in sequence from left to right. A “V” position indicates that the lamp was vertical, “H” that it was horizontal. All measurements were performed in 1994

Day	Date		Measurement Sequence				
1	08–31	Lamp	F-410	OS-27	F-305	F-45	WS-25
		Position	V	H	V	H	V
2	10–19	Lamp	F-410	OS-27	F-305	F-45	WS-25
		Position	V	V	V	V	V
3	10–20	Lamp	F-410	OS-27	F-305	OS-27	F-410
		Position	V	H	V	V	V
4	10–25	Lamp	F-305	F-305	F-305		
		Position	V	H	V		

**Table 2:** Temperatures of lamps in the vertical (*T*_V_) and horizontal (*T*_H_) positions and the temperature differences. The value of *T*_H_ for lamp OS-27 is the average from four determinations, while the values for the other lamps are from one determination each

Lamp	*T*_V_ (K)	*T*_H_ (K)	*T*_V_–*T*_H_ (K)
OS-27	3050.2	3034.5	15.7
F-305	3053.0	3037.3	15.7
F-45	2980.7	2959.4	21.3
